# Editorial: The Phylogenetic History of Hypothalamic Neuromodulators

**DOI:** 10.3389/fnins.2021.712448

**Published:** 2021-06-23

**Authors:** Jackson C. Bittencourt, Giovanne B. Diniz, David A. Lovejoy, Herbert Herzog

**Affiliations:** ^1^Department of Anatomy, Institute of Biomedical Sciences, University of São Paulo, São Paulo, Brazil; ^2^California National Primate Research Center, University of California, Davis, Davis, CA, United States; ^3^Department of Cell and Systems Biology, University of Toronto, Toronto, ON, Canada; ^4^Neuroscience Division, Garvan Institute of Medical Research, Darlinghurst, NSW, Australia

**Keywords:** neurosecretion, peptides, neuromodulators, evolution, diencephalon, invertebrates, vertebrates, homologs

We are delighted to introduce this new Research Topic of “Frontiers in Neuroscience” that focuses on the phylogenetic history and evolution of a number of critical hypothalamic hormones associated with the neuroendocrine regulation of vertebrate energy homeostasis.

The first studies on the concept of neurosecretion were published almost a century ago, beginning with the work of Ernst Scharrer, who showed that the preoptic region of fishes possessed neuroendocrine properties associated with pituitary function. Together with his wife, Bertha, they reported that a neurosecretory system located between the *corpus cardiacum* and *corpus allatum* in insects may be homologous to the hypothalamo-pituitary system of vertebrates. However, it was not until the 1930s that the concept of neurosecretion became integrated into the understanding of how the hypothalamus could play a similar role in vertebrates. Later, Bargmann and Scharrer postulated that vasopressin and oxytocin were produced in the hypothalamus and released via the neural lobe of the pituitary gland. By the 1940s, Geoffrey Harris and John Green established that neuroendocrine factors could flow from the hypothalamus to the pituitary gland. These studies established the modern basis of hypothalamic and pituitary function.

Since then, these seminal studies have spawned advances in comparative neurobiology, endocrinology proteomics, and genomics. However, almost a century after the early studies, we still have much to learn. Our goal in this article collection was not to provide a comprehensive review of the current state of scientific understanding of the multitude of neurohormones associated with the hypothalamus and their role with organismal metabolism and homeostasis, but rather to provide a selective overview of some key studies across a variety of hypothalamic neurohormones and their functions. We anticipate that these publications will foster new research and stimulate new directions in the field of hypothalamus-associated physiology. Given this, we offer several novel articles in various aspects of hypothalamic function.

With respect to the phylogenetic origins of hypothalamic peptides, four studies have focused on the early phylogeny of these peptides. Building upon the initial work on the role of oxytocin and vasopressin release from the neurohypophysis, Odekunle and Elphick have established the early phylogenetic history of oxytocin- and vasopressin-like peptides in invertebrates and their structural relationship to those orthologs in vertebrates. Similarly, but focusing on the adenohypophysis, Cardoso et al. have established that PACAP, a peptide associated with the Secretin family of peptides which possesses structural similarity with peptides found in early evolving metazoans, likely obtained its final gene and peptide form in vertebrates. Another peptide family described in this volume is that of the corticotropin-releasing factor/hormone (CRF/CRH) system. Required for the central regulation of the hypothalalmus-pituitary-adrenal (HPA) axis, and possessing a number of paralogues including CRF2/teleocortin; urocortin/urotensin-I/sauvagine; urocortin 2; and urocortin 3, the CRF system is one of the most ancient hypothalamic systems and serves as a model system to understand how invertebrate molecules evolved into vertebrate neuromodulators. Cardoso et al. from their studies in lampreys, introduce a new and detailed understanding of how CRF gene phylogeny, following the R2 hypothesis, led to the expansion of CRF-related peptides in vertebrates. Finally, Michalec et al. have suggested a novel phylogenetic relationship of secretin, CRF, and insulin-like peptides based on their relationship to the teneurin C-terminal associated peptides (TCAP), which have been suggested to among the earliest peptide precursors to CRF in the metazoans.

Although these studies provide some insight into the first wave of peptides discovered from the earliest studies indicated above, additional new studies provide novel interpretations of vital hypothalamic neuropeptides. Melanin-concentrating hormone (MCH), initially discovered in teleosts, and essential for modulating arousal and energy balance in vertebrates, in addition to numerous other functions, is the subject of a paper by Diniz and Bittencourt. This paper establishes that there are two paralogues of this gene and peptide in teleosts and provides a detailed understanding of these gene duplications and paralogues lead to new functions. Prolactin, likewise, has numerous physiological functions in vertebrates. Dobolyi et al. have provided an evolutionary rationale for the functions of prolactin concerning osmoregulation, growth, and reproduction in vertebrates. The orexins (hypocretins) are among the newer peptides discovered as hypothalamic factors regulating organismal physiology, energy metabolism and, importantly, sleep/wake cycles. Soya and Sakurai provide a novel review of this system with respect to their functions in vertebrates. Finally, due to the essential integratory function of the hypothalamus, most of its critical neurohormonal systems are subject to complex regulation during development. Schredelseker et al. have shown that the brain-specific homeobox transcription factor (Bsx) is required for the transcription of a number of critical energy regulating peptides, including neuropeptide Y, CRF, vasoactive intestinal peptide (VIP), and others, indicating that although these peptides belong to different gene systems, they may be regulated by a common ancestral transcription factor.

We hope the readers will utilize these publications to extend their own research in the structure, function, and phylogeny of these and other hypothalamic modulators.

## Author Contributions

All authors listed have made a substantial, direct and intellectual contribution to the work, and approved it for publication.

## Conflict of Interest

The authors declare that the research was conducted in the absence of any commercial or financial relationships that could be construed as a potential conflict of interest.

